# #KnowMyViralLoad: how community-led demand creation holds a key to routine viral load testing scale-up in Africa

**DOI:** 10.1186/s12889-026-27215-5

**Published:** 2026-04-10

**Authors:** Helen Etya’ale, Collins Otieno Odhiambo, Bactrin Killingo, Susan Perez, Pontsho Pilane, Anafi Mataka, Idrissa Songo, Carol Ameera Kassam, Tonderai Mwareka, Evelyn Letio, Ange Mavula, Nelson Otwoma, Pascale Ondoa

**Affiliations:** 1 Washington University in St Louis, St Louis, USA; 2 African Society for Laboratory Medicine, Addis Ababa, Ethiopia; 3 International Treatment Preparedness Coalition, Johannesburg, South Africa; 4 AIDS Strategy Advocacy and Policy Ltd, Hanoi, Vietnam; 5 Mathaba Media, Johannesburg, South Africa; 6 Network of HIV Positives in Sierra Leone, Freetown, Sierra Leone; 7 Independent Consultant, Lilongwe, Malawi; 8 Zimbabwe National Network of People living with HIV, Harare, Zimbabwe; 9 National Empowerment of Positive Women United, Juba, South Sudan; 10 Union Congolaise des Organisations des Personnes vivant avec le VIH, Kinshasa, Democratic Republic of Congo; 11 National Empowerment Network of People living with HIV/AIDS in Kenya, Nairobi, Kenya; 12 Amsterdam Institute for Global Health and Development, University of Amsterdam, Amsterdam, the Netherlands

**Keywords:** Routine viral load, Community-led initiatives, Digital campaigns, Implementation, HIV

## Abstract

**Background:**

Viral load testing (VLT) is key to detecting treatment failure and is central to HIV management. However, low demand for routine VLT among people on antiretroviral treatment limits opportunities for evidence-based HIV management and transmission reduction. Demand generation for routine VLT can improve through empowering communities of people living with and affected by HIV with relevant, tailored and actionable information on VLT. We sought to identify the most appropriate and context-relevant media platforms and audiences for VLT-related demand generation campaigns in 6 African countries.

**Intervention:**

Two-phase VLT-focused media communication campaigns were conducted in six African countries. We also evaluated the process and outcomes of these campaigns through an anonymized assessment.

**Lessons Learnt:**

We tracked the platform-specific reach of the campaigns, where traditional media platforms like radio and digital media platforms like Facebook, Twitter and WhatsApp had the widest reach. Additionally, respondents to the post-campaign assessment identified how they learnt new information about VLT (Facebook, Twitter, WhatsApp, short messaging services, radio, television, virtual and in-person meetings or peer educators) and provided a rationale for their preferred communication channels. Of all responses (*n* = 558), the most popular reasons were ease of understanding (23%), interactive sharing (19%), fun and entertaining way of presenting (18%) and presentation by a peer (17%). Of 210 post-campaign assessment respondents, 82% credited the campaign for a better understanding of their HIV status, 73% reported getting a VLT, 82% told a friend about VLT, 77% received their results and 73% sought interpretation of their VLT results.

**Conclusions:**

Community-led communication campaigns can effectively generate demand and uptake of routine VLT. Digital platforms, suited to the local context, hold potential for disseminating health messages and improving health seeking action, provided a commensurate effort is made to address laboratory-side barriers to the delivery or uptake of VLT services.

**Supplementary Information:**

The online version contains supplementary material available at 10.1186/s12889-026-27215-5.

## Background

Since 2013, the World Health Organization (WHO) has recommended routine viral load testing (VLT) for people on antiretroviral treatment (ART) to detect and confirm HIV treatment failure –notably at six and 12 months after ART initiation, and every 12 months thereafter [1]. VLT results help clinicians detect non-adherence to ART, provide timely enhanced adherence counselling and confirm treatment failure or viral re-suppression, thus guiding ART management. Achieving an ‘undetectable’ viral load (less than 50 copies/mL), significantly reduces the chance of transmitting HIV to HIV-negative partners and motivates people living with HIV on ART to regularly seek viral load tests [2]. Ensuring widespread access to routine VLT is key to reaching the third pillar of the UNAIDS 2025 95:95:95 targets (achieving viral suppression among 95% of people living with HIV on ART) [3]. However, demand for routine VLT often remains low among people on ART, even in the face of otherwise available VLT capacity [4], thereby limiting opportunities for evidence-based patient management and reduction of HIV transmission within the community. Demand generation for routine VLT can conceivably be improved through empowering communities of people living with and affected by HIV with relevant, tailored and actionable information on viral load testing.

Communities of people living with and affected by HIV have historically played an effective role in mobilizing and successfully addressing barriers to HIV prevention, treatment and care services [5]. More specifically, community-led awareness campaigns have proven to be an effective tool in encouraging the uptake of essential HIV services.

The International Treatment Preparedness Coalition (ITPC), a global activist group comprised of people living with and affected by HIV, has led successful routine VLT-focused demand generation campaigns, and demonstrated how recipients of care and their communities, equipped with the right knowledge, proceed to demand the services they are entitled to [6]. Under the banner of a multi-country campaign entitled *Be Healthy: Know Your Viral Load*, a stepwise process of treatment education on routine VLT and related advocacy actions led to VLT scale-up and highlighted the need for more routine VLT-related research and more accessible VLT technology [6]. 

We sought to determine the unique role of community-led multimedia campaigns to generate demand for routine VLT and improve health-seeking action through a collaboration between ITPC and the Laboratory Systems Strengthening Community of Practice (LabCoP) of the African Society for Laboratory Medicine (ALSM), operating in six African countries.

## Campaign description and rollout

In July to December 2020 (Phase I) and July to December 2021 (Phase II), community organizations developed tailored, country-specific awareness campaigns to address identified routine VLT knowledge gaps using digital platforms. The campaigns were run in six countries: Democratic Republic of Congo (DRC), Kenya, Malawi, Sierra Leone, South Sudan and Zimbabwe. Using the hashtag, #KnowMyViralLoad, the campaigns targeted different audiences including men, women and young people living with HIV as well as expectant mothers, religious leaders, sex workers, and men who have sex with men. The campaign was also run through traditional platforms including print, short messaging service (SMS), TV, radio and peer educators. Prior to the rollout of the campaigns, as part of their routine activities, community organizations conducted rapid consultations within their existing networks of people living with HIV to identify gaps in knowledge related to routine VLT. These consultations highlighted common country-specific misconceptions and information gaps around VLT, including the purpose of the test, recommended testing frequency, and interpretation of results. Phase I, a pilot, sought to identify the topmost context-relevant digital and non-digital media platforms and sub-populations to inform future communication campaigns. In a virtual workshop facilitated by ITPC and ASLM, country teams—made up of representatives from national community organizations, LabCOP and health ministries—developed and disseminated context-specific educational messages. Each country team selected the sub-populations in their local community networks and considered factors such as local languages, literacy, digital media usage patterns and internet access in developing messaging and dissemination strategies. Phase II narrowed down messaging and dissemination to three media platforms and sub-populations in each country, based on reach and engagement data from Phase I, and tracked campaign reach. Table 1 (supplementary file) provides an overview of the target population and selected media per country. Reach and engagement metrics were measured differently for digital media, traditional media and in-person dissemination channels. Reach (unique users exposed) and engagement (likes and reshares) data were obtained from digital platform analytics. Meanwhile, traditional media reach was estimated using broadcast audience estimates provided by media partners, where engagement data collection was not possible. After the nationwide campaigns, in collaboration with ITPC and ASLM, and to obtain preliminary feedback of the campaigns, the community organizations disseminated an anonymized assessment (supplementary file) to their network members who were eligible to receive but had not taken a VLT. Additionally, as the lead local implementation partners, the coalition of community organizations produced reports that detailed the campaign rollout process and provided reflections. Throughout both phases, country teams met regularly online to share best practices and troubleshoot challenges.

Ethical considerations: The post-campaign assessment was conducted by the community organizations in the six countries, with overall program implementation support provided by ITPC, headquartered in South Africa. The assessment was not carried out as a formal research study but as a program evaluation activity. As such, the assessment did not require prospective ethics approval. Nevertheless, all activities were conducted in full compliance with the ethical principles of the Declaration of Helsinki. All participants provided informed consent for their voluntary participation, and data was collected and analyzed anonymously.

## Lessons learnt

**Fig. 1:**
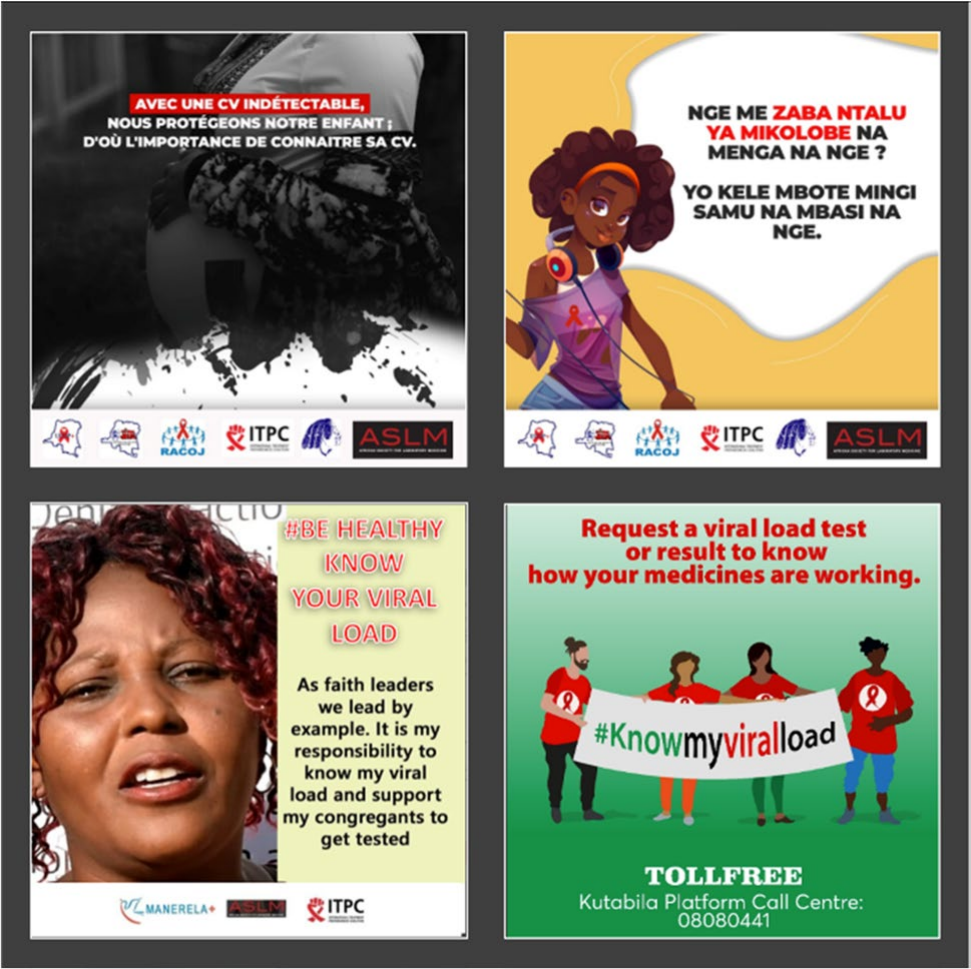
Sample digital campaign flyers: French language flyers in DRC targeting pregnant women (top left); Lingala language flyers targeting youth in DRC (top right), in Malawi targeting religious leaders (bottom left) and in Zimbabwe targeting families (bottom right)

Campaigns were run on the three platforms that generated the most reach and engagement (Phase I) in each country, with messaging targeting select, context-specific audiences. In the DRC, these were WhatsApp, Facebook and peer educators; in Kenya, Facebook, Twitter and virtual peer meetings; in Malawi, WhatsApp, Facebook and peer educators; in South Sudan, Facebook, peer educators and radio; and in Sierra Leone and Zimbabwe, WhatsApp, radio and peer meetings. All countries targeted men and women living with HIV. In addition, the DRC also reached youth and expectant mothers, Kenya included youth and key populations (KPs), Malawi engaged youth and religious leaders, Sierra Leone added KPs and expectant mothers and both South Sudan and Zimbabwe included youth. Overall, across all six countries, the campaigns reached over 100,000 men, women and young people living with HIV as well as expectant mothers, religious leaders, sex workers, and men who have sex with men. The campaigns were run on selected channels, notably Facebook, Twitter, WhatsApp, short messaging services (SMS), radio, television (TV), virtual teleconference and in-person meetings or peer educators. The campaigns had the widest reach among young people, aged 18–24, reaching over 19,000 youth overall. Across all countries, while in-person meetings reached 8582 people, social media platforms like Facebook, Twitter and WhatsApp reached an estimated 22,871, 39,809 and 11,241 people, respectively. The posts that were most shared on social media platforms (Facebook, WhatsApp, and Twitter) generally shared bite-size information in text, image or video format – which increased their appeal and made the information accessible (Fig. 1). Dissemination on other traditional media channels reached an estimated 46,100 people (radio), 7500 people (TV) and 2421 people (SMS).

In addition, across the six countries, a total of 210 people responded to the post-campaign assessment. Respondents listed every channel through which they received new information about routine VLT. Of all the 435 responses, 141 (32%) mentioned in-person meetings with peer educators. WhatsApp (15%), Facebook (13%), radio (11%) and virtual meetings (9%) were the other frequently cited channels. TV (4%) and SMS (3%) were the least mentioned. Respondents also indicated the platforms they found most engaging and effective in helping them learn new information on routine VLT. Of the 198 responses, the majority (50%) preferred in-person meetings although this platform reached less people overall, across all countries. Virtual meetings (11%) and WhatsApp (10%) were the other preferred platforms. Respondents also provided multiple rationales for their choices. Of a total of 558 responses, the most popular reasons were as follows: the information was easy to understand (23%), shared in an interactive way (19%), presented in a fun and entertaining way (18%) and presented by a member of the community (17%).

**Fig. 2:**
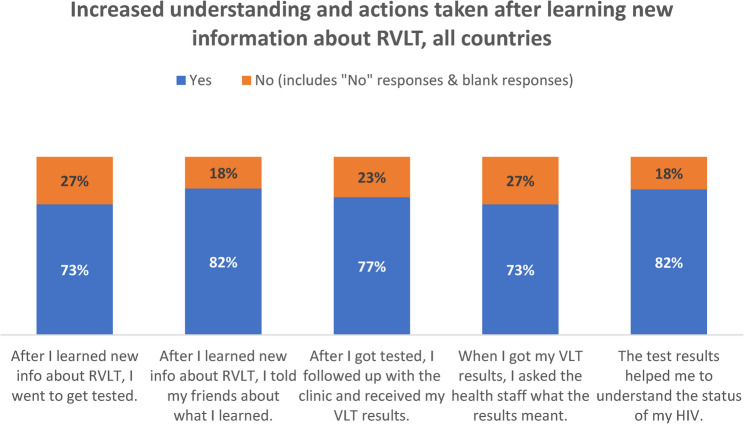
Outcomes of the post-campaign assessment responses in 6 African countries (*n* = 210): increased understanding and health-related action after phase two of the campaigns

Of the 210 post-campaign assessment respondents, 82% credited the campaign for a better understanding of their HIV status. Beyond the knowledge gained, the campaigns also prompted action among respondents, who were eligible but had never taken a VLT: 73% reportedly went to get tested for viral load, 82% told a friend about VLT, 77% received their results and 73% asked a health care staff what the result meant (Fig. 2).

In addition to the campaign assessment, reflections in country program reports highlighted a preference for peer-led, in-person meetings because of the two-way communication and the ability for peer leaders to clarify information. Additionally, the reports also reported instances of VLT testing equipment malfunction and delayed VLT results which dampened the campaign’s demand generation efforts.

## Reflections and recommendations

The importance of community-led awareness campaigns to increase demand for VLT cannot be overstated. This project demonstrated how community-led campaigns can use digital platforms, in addition to traditional communication platforms, to disseminate health messages widely. The post-campaign assessment also revealed the potential for these campaigns to support literacy efforts around routine VLT and trigger health-related action, including seeking a VLT or telling someone about the VLT. Hence, such community-led campaigns bring much value to national HIV program approaches seeking to successfully increase access to HIV services.

However, as was the case in this project, an important paradigm shift is required to enable communities to lead such campaigns in their countries, with the participation of health ministry and laboratory stakeholders. People living with HIV and on treatment are more than mere recipients of services and can play an active role in their health and that of their peers [7]. It is notable that of all the platforms used in this campaign, peer-led, in-person meetings were the most preferred for learning about VLT. The program reports and post-campaign assessment indicated that people valued learning about VLT in ways that were plain, interactive and involved a peer with whom they felt comfortable. In addition to being peer-led, in-person meetings provided a space for interactive exchanges, questions, and clarifications. A foundation of trust, camaraderie and shared experience ensures that the target audience is more receptive to the messaging [8]. Therein lies the unique strength of community-led campaigns: they are designed and implemented by members of the community of people living with and affected by HIV themselves.

This project also demonstrated how digital media platforms can amplify community-led campaigns through wider and more tailored reach. The campaign assessment insights suggest that digital communication campaigns are feasible within the African context. Recent civil society movements have demonstrated the power of the internet for learning, advocacy and demand creation [9] Additionally, internet penetration rate in Africa is estimated at 40%, and the proportion of African internet users increased from about 4% of the global total in 2009 to 13% in 2021 [10]. Digital campaigns, therefore, provide a viable alternative when in-person gatherings are not ideal or are limited, due to HIV-related stigma and discrimination, or not possible, as was the case during the acute phases of the COVID-19 pandemic. In addition, tailored digital community-led campaigns are particularly effective in reaching sub-sections of the population. Our campaign had the widest reach among young people, suggesting that digital platforms can serve as additional avenues to engaging young people–a viable option in sub-Saharan Africa, given the predominantly young and digitally literate African population [11]. Digital campaigns are powerful, provided they are informed by a good understanding of the local context. Hence, this will entail a robust assessment of country routine VLT gaps, demographics, smartphone access, digital literacy levels and the local IT infrastructure. This underscores the value of Phase I, running the campaigns and selecting the most engaging and contextually relevant platforms in each country.

Implementing these campaigns enabled multistakeholder collaboration that brought together health ministry representatives, members of the LabCOP network and community group representatives, who worked collaboratively in-country and exchanged ideas with teams across countries. Hence, while the campaigns were driven primarily by local community entities, this partnership ensured that the initiatives were aligned with and integrated within national HIV programming. Such partnerships provide a model worth exploring for future, sustainable and community-led campaigns within and between African countries.

While the campaigns raised awareness of the importance of VLT, their potential impact on generating demand for testing may have been limited by systemic barriers to VLT, including laboratory reagent stockouts, machine breakdowns and long turnaround times for test results. This may lead to increased demand for VLT without the corresponding supply of viral load tests, dampening a campaign’s impact on the community. Consequently, successful community demand campaigns are useful in generating demand for VLT, but their impact will be limited without concurrent laboratory systems strengthening initiatives. Beyond the campaign implementation, our post-campaign assessment had a few limitations. Due to the inability to poll people reached through traditional and digital media, the campaign relied on voluntary responses from members of community networks who were exposed to the campaigns, potentially introducing response bias. Additionally, collecting baseline viral load test awareness as well as disaggregating data per country would strengthen the post-campaign assessment. Future assessments can build on emerging insights and further explore these questions with a larger and more representative sample of the campaign audience.

## Conclusion

Community-led demand generation campaigns are a powerful tool in our efforts to increase demand and uptake of routine VLT. In addition to traditional media and in-person events, this project demonstrated the potential of communication campaigns vehiculated through digital platforms to increase reach, influence HIV treatment literacy and improve related health-seeking action. However, tailored messaging appropriate to the targeted audience and the preferred channel of communication must be considered in the design of community-led campaigns to maximize their reach and impact. Moreover, the design of these campaigns must also include other relevant stakeholders including the health ministries, laboratory technicians and clinicians to anticipate future demand and ensure the necessary laboratory infrastructure is in place to support VLT uptake.

## Supplementary Information

Supplementary Material 1.

Supplementary Material 2.

## Data Availability

The raw data and analyses can be made available, upon request.
