# Supplementary material for: #KnowMyViralLoad: how community-led demand creation holds a key to routine viral load testing scale-up in Africa
Source: BMC Public Health. 2026 Apr 10;26:1642. doi: 10.1186/s12889-026-27215-5 (PMC13195822; doi:10.1186/s12889-026-27215-5)
Supplement: Supplementary file 1 — Supplementary Material 1. [file 12889_2026_27215_MOESM1_ESM.docx]

**RVLT CAMPAIGN ASSESSMENT QUESTIONNAIRE**

**1.IDENTIFYING INFORMATION – please indicate the last four digits of your telephone number (please rest assured that is only used as a code identifier):**

**2. In case of need for any follow up questions, please indicate if you agree to be contacted by the ASLM country staff and/or ITPC.**

€ Yes, I agree to be contacted, if needed. Please provide an email address or telephone number here (including country code):

€ No, I do not want to be contacted

**3. In the last three months, I learned new information about routine viral load testing (RVLT) through the following: please check all the apply**

€ Facebook

€ Twitter

€ WhatsApp

€ SMS

€ Radio

€ TV

€ Virtual meeting/event (not connected to social media)

€ In-person/support meeting/peer educators

€ I did not learn new information about RVLT from any of these sources. If you check this box, please complete Part 4 only.

€ Other. Please specify:

**4. A viral load test is a measurement of the amount of HIV virus in a sample of blood.**

€ Yes

€ I don’t know

**5. If you get a viral load test and your results say your viral load is “undetectable”, this means (Select ONE correct answer):**

€ Your viral load is greater than 1000 copies/µl

€ Your viral load is (less) 50 or 40 or 20 copies/µl, depending on the machine you are using

€ There is no such thing as an “undetectable” viral load

**6. If I take my medication every day and my viral load is “undetectable” for 6 months, it means that I’m unlikely to transmit HIV to my sexual partners.**

€ Yes

€ No

€ Not sure

**7. I know where to test for my viral load.**

€ Yes

€ No

€ Not sure

**8.Out of the new information I learned from the sources listed in Part 3 (question 1), which method/platform was the most effective or engaging in helping me to learn new information? Please check only ONE campaign platform.**

€ Facebook

€ Twitter

€ WhatsApp

€ SMS

€ Radio

€ TV

€ Virtual meeting/event (not connected to social media)

€ In-person/support meeting/peer educators

€ Other. Please specify:

**9. Based on your response to the previous question (8), why was the method/platform effective or engaging? Please check all that apply.**

€ The information was easy to understand.

€ The information was shared in an interactive way and I could ask questions.

€ The information was presented in a fun, entertaining way so it kept my attention.

€ The information was presented by people from my community so I felt comfortable.

€ The information related to my specific situation.

€ The information was not too much and not too overwhelming.

€ Other. Please explain:

**10. After I learned new information about RVLT (see responses to Part 3), I went to get tested.**

€ Yes

€ No

**11. After I learned new information about RVLT (see responses to Part 3), I told my friends about what I learned.**

€ Yes

€ No

**12. After I got tested, I followed up with the clinic and received my viral load test results.**

€ Yes

€ No

**13. When I got my viral load test results, I asked the health staff what the results meant.**

€ Yes

€ No

**14.The test results helped me to understand the status of my HIV.**

€ Yes

€ No

**15. Since I got tested, I’ve shared with others about my experience getting a viral load test.**

€ Yes

€ No
