# Supplementary material for: #KnowMyViralLoad: how community-led demand creation holds a key to routine viral load testing scale-up in Africa
Source: BMC Public Health. 2026 Apr 10;26:1642. doi: 10.1186/s12889-026-27215-5 (PMC13195822; doi:10.1186/s12889-026-27215-5)
Supplement: Supplementary file 2 — Supplementary Material 2. [file 12889_2026_27215_MOESM2_ESM.docx]

**Supplementary Table 1: Community partners, target audiences and primary dissemination platforms used in the #KnowMyViralLoad campaigns**

| **Country** | **Community organisations involved** | **Target audiences** | **Primary**  **dissemination platforms** |
| --- | --- | --- | --- |
| Democratic Republic of Congo | Union Congolaise des Organisations des PVVIH (UCOP)+  Réseau des Associations Congolaises des Jeunes (RACOJ)  Fondation Femme Plus (FFP)  Programme National de Lutte contre le Sida | Adult men and women  Youth  Expecting mothers | WhatsApp  Facebook  Peer educators |
| Kenya | National Empowerment Network of People Living with HIV and AIDS in Kenya (NEPHAK)  Positive Young Women’s Voices (PYWV)  Ambassador for Youth and Adolescent Reproductive Health (AYARHEP)  International Community of Women Living with Women (ICW - Kenya) | Adult men and women  Youth  Key populations | Facebook  Twitter  Virtual peer meeting |
| Malawi | Malawi Network of Religious Leaders Living with or affected by HIV/AIDS (MANARELA+)  Coalition of Women living with HIV/AIDS  YPLUS | Adult women  Youth  Religious leader | WhatsApp  Facebook  Peer educators |
| Sierra Leone | Network of HIV Positives in Sierra Leone (NETHIPS)  Happy Kids and Adolescent  National AIDS Control Programme  Dignity Association (MSM), SWAASL, WICM (FSW) | Adult men and women  Expecting women  Key populations | WhatsApp  Radio  Peer educators |
| South Sudan* | National Empowerment of Positive Women United (NEPWU) | Adult men  Adult women  Youth | Facebook  Peer educators  Radio |
| Zimbabwe* | Zimbabwe National Network of People Living with HIV  Zimbabwe Young Positives | Adult men  Adult women  Youth | WhatsApp  Peer educators  Radio |

*South Sudan and Zimbabwe considered adult men and women as separate populations
